# Commentary: Neural correlates of expected risks and returns in risky choice across development

**DOI:** 10.3389/fnhum.2015.00388

**Published:** 2015-06-30

**Authors:** Faisal Mushtaq, Liam J. B. Hill, Amy R. Bland, Matt Craddock, Neil B. Boyle

**Affiliations:** ^1^School of Psychology, Faculty of Medicine and Health, University of LeedsLeeds, UK; ^2^Neuroscience and Psychiatry Unit, University of ManchesterManchester, UK

**Keywords:** decision making, risk-taking, development, fMRI, neuroscience of decision making, cognitive development, neuroeconomics

“Wisdom comes with age” is an oft-heard expression. It suggests that across development we improve in our ability to make decisions—but evidence for its validity is equivocal. In real-world decision-making, there is an adolescent-specific increased propensity to engage in behaviors associated with morbidity and mortality (e.g., road traffic accidents, unprotected sex, violence, drug, and alcohol abuse; Blum and Nelson-Mmari, 2004). However, this inverted u-shape developmental trajectory for risk-taking is typically not observed in laboratory-based studies (Defoe et al., 2015). As such, there exists a need to: (a) bridge the gap between laboratory and real world behavior; and (b) clarify the processes underlying developmental differences in decision-making to inform interventions that target a reduction in health-risking adolescent activities.

A recent fMRI study by Van Duijvenvoorde et al. (2015) examined the neural correlates of risk-taking in three maturational periods: childhood, adolescence, and adulthood. Participants from each age group performed a version of the Columbia Card Task (CCT; Figner et al., 2009). In the fMRI-CCT participants are shown up to 16 cards face down per round—each card is associated with a gain or loss of reward and participants are informed about the proportion of cards associated with loss and gain during each round. Participants turn over cards until they either decide to stop or until they turn over a loss card, which ends the round. Loss probability increases with each card selection—as each gain card drawn reduces the proportion of gain cards in the remaining set. This follows a “Risk-Return” approach to understanding decision-making. In economics these tasks are used to isolate risky decisions into two components: (i) a return—which provides a measure of the expected value of the choice; and (ii) a risk component—which provides an index of outcome variability (e.g., SD of possible outcomes; Mushtaq et al., 2013). The degree to which a participant balances the potential *reward* for turning over another card with the potential *risk* allows a clear characterization of an individual's risk-taking behavior (Weber, 2010). This model, novel to developmental neuroimaging studies, has a clear advantage over classic utility-based models (e.g., tasks centered around prospect theory), which have difficulty in parsing out the contributions of risk and reward in decision-making. This is crucial for linking neural data to underlying cognitive processes as it allows researchers to tease out differences in risk and return sensitivity along the developmental pathway. Indeed, many previous imaging studies on developmental differences in the neural substrates of risk-taking confound these components (see Richards et al., 2013 review)—leading to an impasse in our understanding of how risk processing alone might vary over development. van Duijvenvoorde et al.'s study directly tackles this issue.

The ultimate goal of neuroimaging is to relate neural processes to behavior, but frequently fMRI analysis is limited to the production of statements regarding the relative magnitude of distinct conditions. Van Duijvenvoorde et al. (2015) study is an example of the value that can be obtained from mapping the relationship between brain and behavior. The decomposition of task performance into risk- and return-sensitivity allowed the identification of distinct areas that parametrically coded risk and return. Another interesting take on data analysis was the use of linear and quadratic models. The authors reasoned that if developmental changes followed a monotonic pattern over time, a linear model could describe them. If the effects were asymmetrically sensitive to adolescence specific effects, a quadratic function would best fit the data. These innovative approaches, through task employed and analysis procedure, allowed the authors to elucidate the specific neural mechanisms underlying the full dynamic of risky decision-making across differential maturational stages.

The results show the independence of reward and risk related cognitive processes in neural circuitry and patterns of maturation. Specifically, the researchers observed heightened activation for risk in adolescents in regions previously associated with risk processing, namely the right anterior insula, inferior frontal gyrus and the dorsal medial PFC (consistent with Preuschoff et al., 2008; Mohr et al., 2010). Importantly, adolescents' activations in these regions significantly correlated with their risk-sensitivity. In contrast, return related activity increased linearly with age in ventral medial PFC and posterior cingulate cortex (PPC) and correlated positively with behavioral return sensitivity. This approach provides two critical insights into developmental differences in decision-making hitherto unknown and would be difficult to parse out with traditional decision-making tasks: (1) they show qualitatively different developmental changes in processing of risk and reward; and (2) fMRI results demonstrate a heightened adolescent specific response to the processing of risk.

These findings are an important first step and give rise to a number of interesting questions for future research. For example, in van Duijvenvoorde et al.'s study the individual variability in risk propensity was amplified in the adolescent group. To account for similar inter-individual variability, decision scientists have increasingly begun to focus on individual risk-preference and traits as decision predictors- ideologically moving from a nomothetic to idiographic approach (Stanovich and West, 2000). In other words, there has been an increased focus on individual risk-preference and traits as decision predictors alongside, or instead of, aggregate group-level analysis. Relatedly, another recent study (Gilaie-Dotan et al., 2014) reported gray matter volume in the right PPC can predict an individual's risk attitude. This may well-serve as a neural biomarker for risk propensity. Whilst such results are not causal in nature, they may provide a means of accounting for some proportion of the differences in adolescence. Convergence between such structural and functional neural evidence offers considerable scope for future research.

To summarize, Van Duijvenvoorde et al.'s (2015) study provides novel insights into the neural mechanisms of reward and risk sensitivity across maturation. Importantly, it delineates the neural circuitry involved in the etiology of risk processing and in doing so uncovers age-related neural differences that, with further investigation, may help explain variability in risk-propensity across the lifespan. Given the financial and health impact of sub-optimal risk seeking, being able to identify associations between brain, development and behavior holds promise to improve risk-avoidance interventions, thus providing substantial economic and societal benefit.

## Conflict of interest statement

The authors declare that the research was conducted in the absence of any commercial or financial relationships that could be construed as a potential conflict of interest.
